# A Juvenile Story

**Published:** 1886-08

**Authors:** 


### *A JUVENILE STORY.*

### HOW IS THIS FOR A TWELVE-YEAR-OLD VERMONT GIRL.

The Rutland *Herald* gives the following school composition which was written by a little Vermont girl twelve years old. It is said to be given just as it was written without correction. She was only half an hour in writing it and had no book of reference. She told her papa, however, that she had thought it well out the night before:

*A Story of a Red Blood Corpuscle.*—As I am resting in a piece of tissue to which I was sent, I thought I might as well write my adventures, which are many and varied. I first came to life with many of my relations in a large fleshy room which I came to know was the right ventricle of the heart, then by a sudden squeeze we were sent altogether into a long canal with hard walls (it was the pulmonary artery) there were so many of us that we were all jammed together, and I said to myself, "I had a great deal rather go back into that nice large room again," so I ran back as fast as I could, but lo and behold, three little doors barred my way, and the more I pushed the tighter they closed, and so I gave it up and went back to my fellows. We then went on into smaller veins or capillaries, and through thin walls. I could see the spongy substance called the lungs. On we travelled into an immense vein called the pulmonary vein, and from there into a smaller room than the one we were in first; presently another squeeze sent us from the left auricle, as it is called, into the left ventricle, and from there into another large artery called the aorta; just then I and my companions were startled by a telegraphic message from the brain, "We are busy with a Latin verb and need red blood corpucles to help us"; about 2,000,000 of us started for the brain, but we had hardly got half way before there came a dispatch from the arms, "We have been swinging clubs and have used a great deal of tissue. Come and help us." Half of us went on to the brain while the other went to the arm. And here I am expecting to be used up any minute. Ah, here I go! the arm has raised and I must die.

